# Stereochemistry of the methyl­idene-bridged quinazoline-iso­quinoline alkaloid 3-{[6,7-dimeth­oxy-1-(4-nitro­phen­yl)-1,2,3,4-tetra­hydro­isoquinolin-2-yl]methyl­idene}-1,2,3,9-tetra­hydro­pyrrolo­[2,1-*b*]quinazolin-9-one methanol monosolvate

**DOI:** 10.1107/S2056989020006696

**Published:** 2020-05-22

**Authors:** Akmal Tojiboev, Sherzod Zhurakulov, Valentina Vinogradova, Ulli Englert, Ruimin Wang

**Affiliations:** a Laboratory of Thermophysics of Multiphase Systems, Institute of Ion-Plasm and Laser Technologies named after U.A. Arifov, Academy of Sciences of Uzbekistan,100125, Durmon yuli st. 33, Tashkent, Uzbekistan; b S.Yunusov Institute of Chemistry of Plant Substances, Academy of Science of, Uzbekistan, Mirzo Ulugbek Str. 77, 100170 Tashkent, Uzbekistan; c Institute of Inorganic Chemistry, RWTH Aachen University, Landoltweg 1, 52056, Aachen, Germany

**Keywords:** quinazoline, iso­quinoline, steric congestion, hydrogen bonding, crystal structure

## Abstract

Two potentially bioactive fragments, namely a tricyclic quinazoline derivative with an exocyclic alkene moiety and a substituted iso­quinoline, are coupled to give 3-{[6,7-dimeth­oxy-1-(4-nitro­phen­yl)-1,2,3,4-tetra­hydro­isoquinolin-2-yl]methyl­idene}-1,2,3,9-tetra­hydro­pyrrolo­[2,1-*b*]quinazolin-9-one. The target product crystallizes as a methanol solvate, C_29_H_26_N_4_O_5_·CH_4_O, and is *E* configured. The alternative *Z* isomer would necessarily imply either considerable twist about the central double bond or very unfavourable intra­molecular contacts between sterically more demanding substituents. The main residue and the co-crystallized solvent mol­ecule aggregate to discrete pairs *via* a classical O—H⋯O hydrogen bond with a distance of 2.8581 (7) Å between the methanol OH donor and the quinazolinone O=C acceptor.

## Abstract 2

The main residue and the solvent mol­ecule aggregate to discrete pairs *via* a classical O—H⋯O hydrogen bond, while non-classical C—H⋯O inter­actions lead to the formation of an extended network.

## Chemical context   

**Figure 1:**
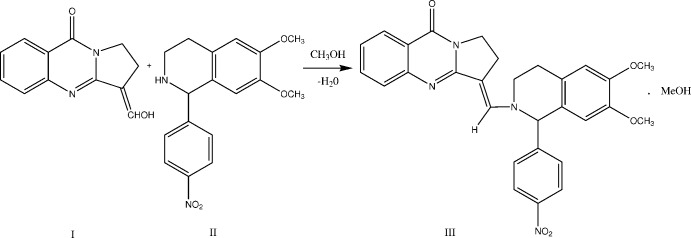
Chemical scheme showing the synthesis of the title compound

The synthesis of the title compound, 3-[1′-(4′′-nitro­phen­yl)-6,7-dimeth­oxy-1,2,3,4-tetra­hydro­iso­quinoline-2-yl)]-methyl­idene-1,2,3,9-tetra­hydropyrrolo­[2,1-*b*]quinazolin-9-one meth­anol solvate, (III) is shown in Fig. 1 ▸. It combines two well-known bioactive scaffolds, namely a tricyclic quinazoline derivative (I) and a substituted iso­quinoline (II).

Tricyclic quinazoline alkaloids are frequently encountered in nature (Michael, 1997 ▸; Eguchi, 2006 ▸; Shakhidoyatov *et al.*, 2014 ▸). The reason for the wide inter­est in studying these substances lies in their multi-facetted biological activity: they have been associated with anti­bacterial (Jantova *et al.*, 2004 ▸), tumor growth-inhibiting (Aoyagi *et al.*, 1999 ▸; Kuneš *et al.*, 2000 ▸; Foster *et al.*, 1999 ▸; Forsch *et al.*, 2002 ▸; Abdel-Jalil *et al.*, 2005 ▸), anti­fungal (Dandia *et al.*, 2005 ▸; Nikhil *et al.*, 2011 ▸), anti­hyperglycemic (Ram *et al.*, 2003 ▸) and anti-inflammatory (Yeh-Long *et al.*, 2004 ▸) activity. They have been used as a bronchodilator (Jindal *et al.*, 2002 ▸), cholinesterase inhibitor (Decker, 2005 ▸), anti­folate (Rosowsky *et al.*, 2000 ▸) and as a protein kinase inhibitor (Levitzki *et al.*, 2003 ▸). Additional reports suggest these derivatives are used as anti-cancer (Manoj *et al.*, 2013 ▸), anti-HIV (Zaigang *et al.*, 2009 ▸), anti­convulsant and anti­hypertensive (Muruganantham *et al.*, 2004 ▸) drugs and as anti­oxidants (Srinubabu *et al.*, 2014 ▸). The Cambridge Structural Database (CSD, version 5.40, update February 2019; Groom *et al.*, 2016 ▸) contains 118 structurally characterized substituted tricyclic quinazolines. Different methods for their efficient synthesis have been developed (Bowman *et al.*, 2007 ▸; Deetz *et al.*, 2001 ▸; Kamal *et al.*, 2001 ▸, 2004 ▸; Lee *et al.*, 2003 ▸; Liu *et al.*, 2005 ▸). The reactive centres in the tricyclic quinazoline scaffold allow for further derivatization *via* electrophilic or nucleophiles substitution.

Iso­quinoline alkaloids represent a particularly popular and widespread group of alkaloids. Even for fairly simple iso­quinoline derivatives, biological activity has been reported. Examples include analgetic, anti-inflammatory and anti-cancer properties (Jeetah *et al.*, 2014 ▸), anti-AIDS (Uesawa *et al.*, 2011 ▸), anti­fungal activity (Kashiwada *et al.*, 2005 ▸) and cardiovascular effects (Candenas *et al.*, 1990 ▸). Antagonists for the pathogenesis of neurological diseases, such as Parkinson’s disease (Zaima *et al.*, 2012 ▸) have also been described. A group of synthetic 1-aryl­tetra­hydro­iso­quinoline derivatives show anti­epileptic (Gitto *et al.*, 2003 ▸), analgesic (Tursunkhodzhaeva *et al.*, 2012 ▸) and sedative-anxiolytic activity (Mirzaev *et al.*, 2017 ▸).

Over the years the synthetic inter­est in the quest for new iso­quinoline derivatives has not declined (Bentley, 2006 ▸; Zhurakulov *et al.*, 2013 ▸, 2014 ▸, 2015 ▸), because even minor changes in the mol­ecular geometry may lead to improved therapeutic effects. Both moieties mentioned above, a quinazoline and an iso­quinoline, have been successfully connected by a methyl­idene bridge (Elmuradov *et al.*, 1998 ▸, 2008 ▸; Turdibayev *et al.*, 2011 ▸; Zhurakulov *et al.*, 2015 ▸). This coupling reaction allows two potentially bioactive components to be combined in a single mol­ecule. In view of the high chemical and biological activity of iso­quinoline and tricyclic quinazoline alkaloids, we expect that the combination of both scaffolds as in the target compound of the present study could lead to unprecedented properties.
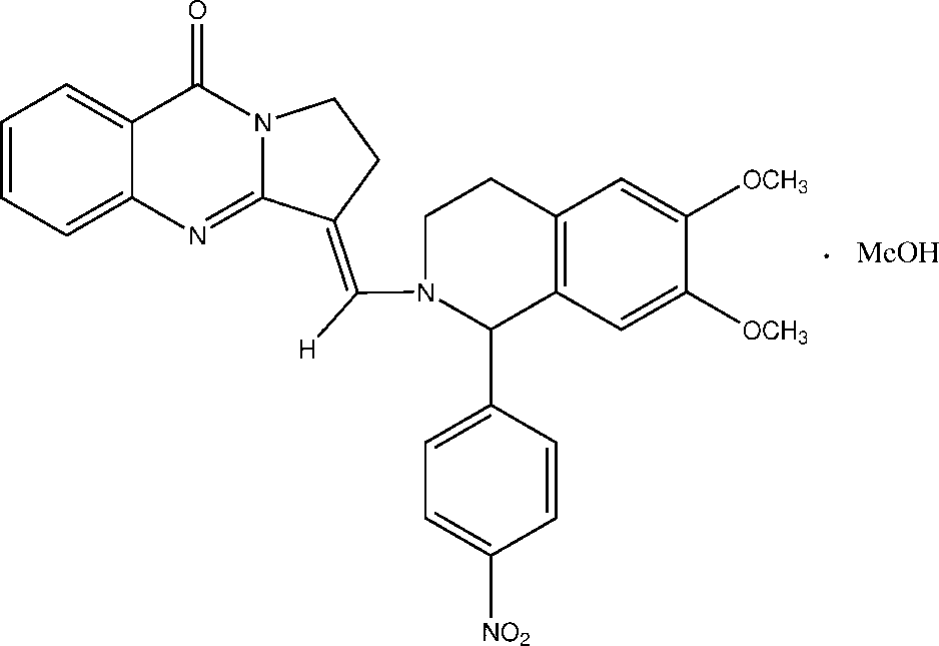


## Structural commentary   

**Figure 2:**
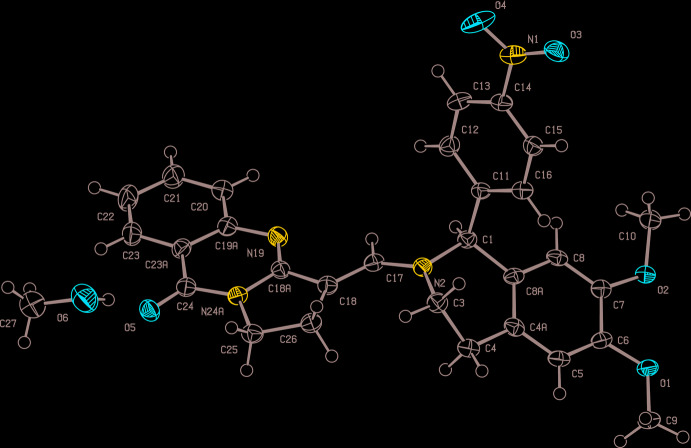
Displacement ellipsoid plot (Spek, 2020 ▸) of the asymmetric unit of 3-[1′-(4′′-nitro­phen­yl)-6,7-dimeth­oxy-1,2,3,4-tetra­hydro­isoquinol-2-yl)]methyl­idene-1,2,3,9-tetra­hydro­pyrrolo­[2,1-*b*]quinazolin-9-one with the methanol solvate and atom-labelling scheme. Ellipsoids are drawn at 50% probability, H atoms are shown as spheres of arbitrary radius.

The title compound crystallizes in the monoclinic space group *P*2_1_/*n* with one mol­ecule of the target heterocycle and one mol­ecule of methanol in the asymmetric unit. A displacement ellipsoid plot and the numbering scheme for both mol­ecules are provided in Fig. 2 ▸.

**Figure 3:**
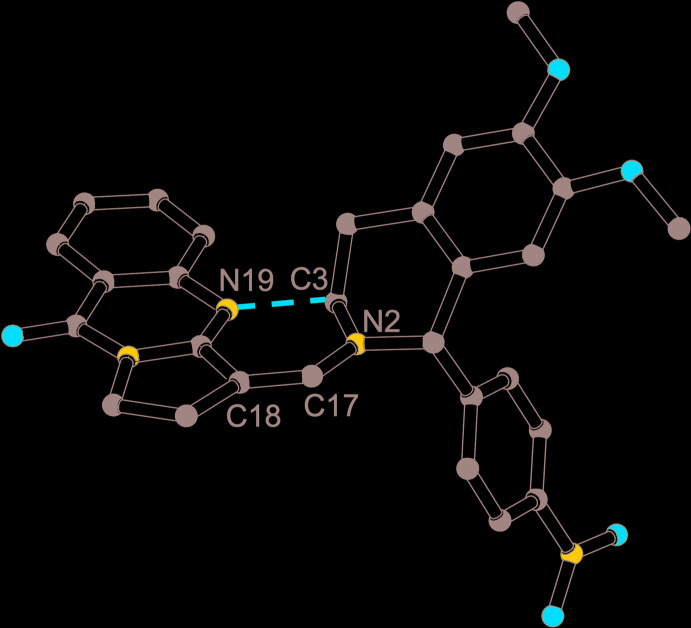
Ball and stick representation (Spek, 2020 ▸) of a hypothetical *Z-*configured mol­ecule generated by 180° rotation of all atoms of the tricyclic quinazoline moiety about the C17=C18 bond; the dashed red line emphasizes the unfavourable intra­molecular contact (see text).

The meth­oxy substituents associated with O1 and O2 are displaced slightly out of the mean plane defined by the aromatic ring in the di­hydro­iso­quinoline moiety (C4*A*–C8*A*), with out-of-plane distances of 0.082 (3) Å for C9 and 0.221 (3) Å for C10. The twist conformation of the heterocyclic ring of the di­hydro­iso­quinoline moiety and the equatorial position of the nitro­phenyl substituent observed here are similar to those in related structures (Olszak *et al.*, 1996 ▸; Turgunov *et al.*, 2016 ▸). C1, C4, C4*A* and C8*A* are coplanar within error, whereas C3 and N2 are on opposite sides of this plane. The nitro­phenyl substituent C11–C16 and the aromatic part of the di­hydro­iso­quinoline (C4*A*–C8*A*) form an angle of 75.70 (14)°. The main motivation for our crystallographic study was to establish the configuration about the C17=C18 double bond. Intuition suggests that the *E* configuration should clearly be favoured, and our experiment confirms this expectation. In order to further explore the steric congestion of an alternative *Z* configuration, we generated such a hypothetical mol­ecule by 180° rotation of the complete tricyclic quinazoline moiety about C17=C18. The resulting geometry is depicted in Fig. 3 ▸.

The prohibitively short intra­molecular contact between N19 and C3, shown as a dashed red line, amounts to only 2.05 Å without taking the hydrogen atoms attached to C3 into account. If the two parts of the target mol­ecule are perceived as at least moderately rigid groups, such an alternative *Z* configuration can safely be excluded. It is important to note, however, that this construction of a hypothetical *Z-*configured mol­ecule relies on the experimentally established geometry of the semi-rigid iso­quinoline and quinazoline moieties. The tricyclic quinazoline system, formed by three fused rings, shows deviations from planarity for the *sp* ^3^ carbon atoms, with maximum displacements of 0.126 (3) Å for C26 and 0.110 (3) Å for C25 on opposite sides of the mean plane.

## Supra­molecular features   

**Figure 4:**
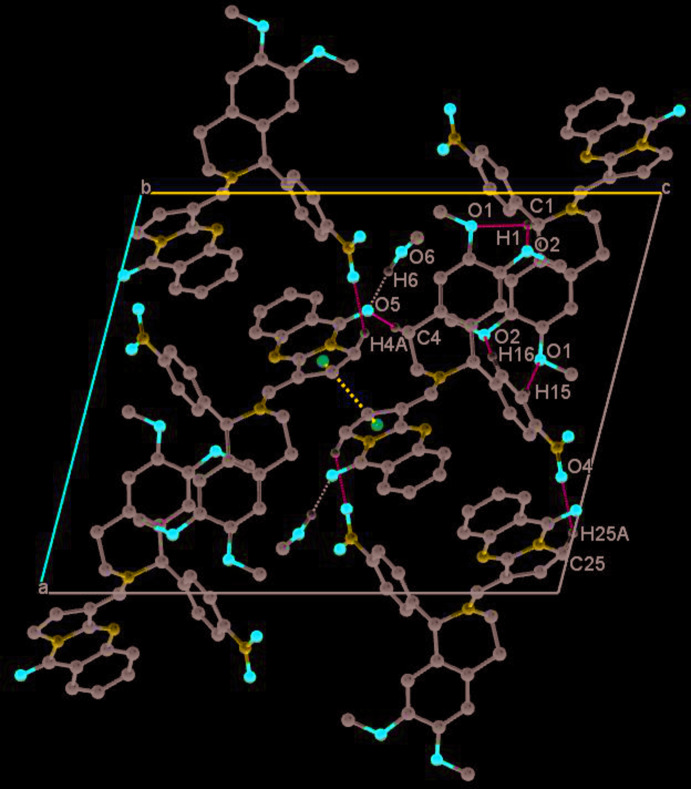
Crystal packing in a view along the *b* axis. O—H⋯O bonds are shown as black, C—H⋯O contacts as blue dashed lines. The dark-blue dotted line indicates a stacking inter­action.

An O⋯H—O hydrogen bond links the co-crystallized methanol mol­ecule to the keto group of the quinazoline moiety and gives rise to a *D*(2) graph-set motif (Table 1 ▸). Additional short contacts involve non-classical C—H⋯O inter­actions, with H⋯O distances ranging between 2.29 and 2.59 Å, forming a complex three-dimensional network (Table 1 ▸, Fig. 4 ▸).

**Figure 5:**
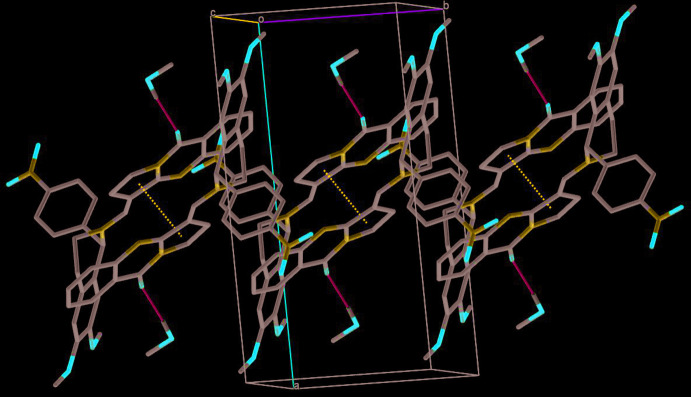
View approximately along the *c* axis, showing stacking between the pyrrole rings (dashed dark-blue lines). The O—H⋯O hydrogen bond is shown in light blue, other hydrogen atoms have been omitted.

Stacking (Fig. 5 ▸) occurs between the pyrrole rings of neighbouring mol­ecules about a centre of inversion [symmetry code: (i) 1 − *x*, 1 − *y*, 1 − *z*], with a distance between the centroids *Cg*1⋯*Cg*1^i^ of 3.832 (2) Å and a ring slippage of 1.246 Å. Both short inter­molecular contacts together lead to a supra­molecular layer structure parallel to the (010) plane.

## Hirshfeld surface analysis   

**Figure 6:**
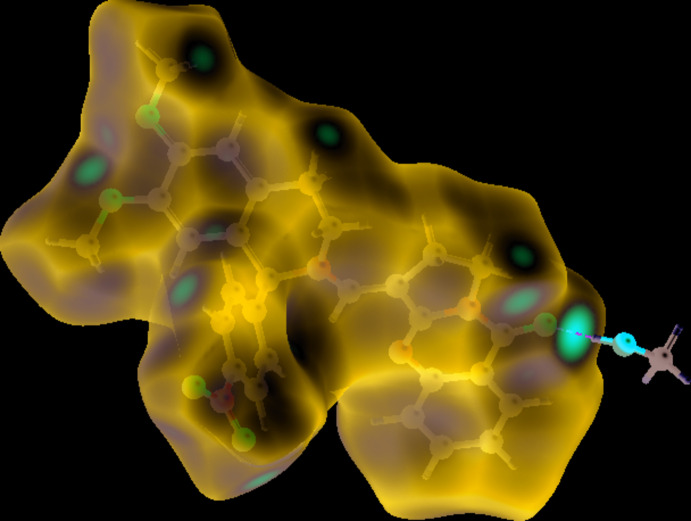
View of the three-dimensional Hirshfeld surface of III mapped with *d*_norm._

**Figure 7:**
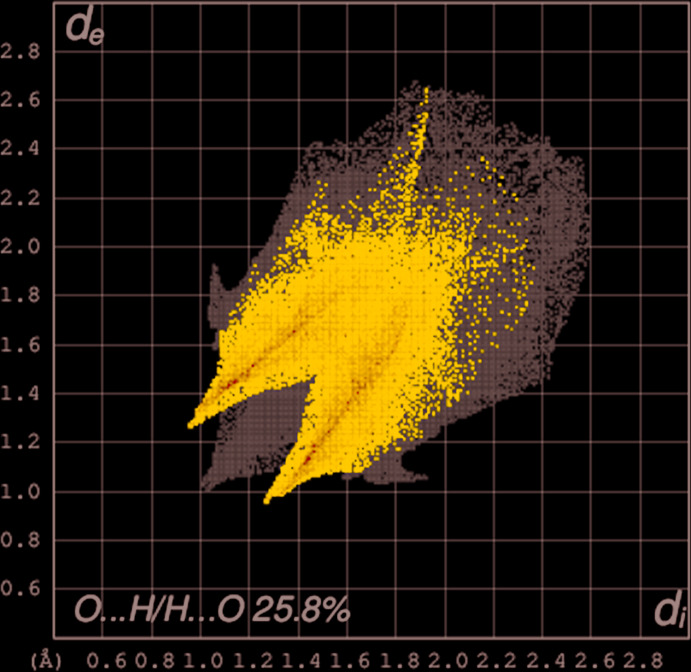
Two-dimensional fingerprint plots for III, showing O⋯H/H⋯O inter­actions. The *d*_i_ and *d*_e_ values are the closest inter­nal and external distances (in Å) from given points on the Hirshfeld surface contacts.

The Hirshfeld surface analysis (Spackman & Jayatilaka, 2009 ▸) and the associated two-dimensional (2D) fingerprint plot (McKinnon *et al.*, 2007 ▸) were performed with *CrystalExplorer17* (Turner *et al.*, 2017 ▸). The Hirshfeld surface for the main mol­ecule in III, mapped with *d*_norm_ and its inter­action with the co-crystallized solvent mol­ecule is represented in Fig. 6 ▸. Colours on the Hirshfeld surface encode contact distances (red - close, white - medium, blue - long) between atoms on either side of the surface. The most obvious inter­molecular inter­action, the classical O⋯H—O hydrogen bond, shows up as a prominent deep-red spot on the surface, oriented towards the co-crystallized methanol mol­ecule. The less-pronounced red features on the surface are associated with C—H⋯O contacts. Fig. 7 ▸ shows a 2D fingerprint plot for the contacts between O and H atoms. These contacts are responsible for the short lateral ‘spikes’ on either side of the main diagonal of the plot.

## Database survey   

A search in the Cambridge Structural Database (CSD, version 5.40, update February 2019; Groom *et al.*, 2016 ▸) gave seven occurrences of mol­ecules containing the 3-methyl­idene-1,2,3,9-tetra­hydro­pyrrolo­[2,1-*b*]quinazolin-9-one moiety with a similar planar conformation as in the title structure. A search for the 1′-(4′′-nitro­phen­yl)-6,7-dimeth­oxy-1,2,3,4-tetra­hydro­iso­quinoline-2-yl moiety gave only three hits with similar conformations for the iso­quinoline fragment: 1-(4-nitro­phen­yl)-*N*-(2,3,4,6-tetra-*O*-pivaloyl-β-d-galacto­pyranos­yl)-6,7-di­meth­oxy-1,2,3,4-tetra­hydro­iso­quinoline (ABUTIA01; Allef *et al.*, 2007 ▸) and two additional structures with a chloro-substituted phenyl ring, namely 2-acetyl-1(*R*)-(4′-chloro­phen­yl)-6,7-dimeth­oxy-1,2,3,4-tetra­hydro­iso­quinoline (ADOCUS; Gitto *et al.*, 2007 ▸) and *N*-acetyl-1-(4-chloro­phen­yl)-6,7-dimeth­oxy-1,2,3,4-tetra­hydro­iso­quinoline (LEFFIM; Gao *et al.*, 2006 ▸).

## Synthesis and crystallization   

3-Hy­droxy­methyl­idene-1,2,3,9-tetra­hydro­pyrrolo­[2,1-*b*]quinazolin-9-one (I) was synthesized according to the method of Oripov *et al.* (1979 ▸). Compound III was obtained from reaction of 1-(4′-nitro­phen­yl)-6,7-dimeth­oxy-1,2,3,4-tetra­hydro­iso­quinoline (0.164 g, 0.522 mmol) with 3-hy­droxymethyl­idene-1,2,3,4-tetra­hydro­pyrrolo­[2,1-*b*]-quinazolin-9-one (0.122 g, 0.522 mmol). Yield 0.22 g, 86%; m.p. 462–465 K (after crystallization from methanol), R_f_ 0.81 (CHCl_3_/MeOH 14:1). A detailed report on the synthesis of III and its characterization by NMR, IR and mass spectrometry is available (Zhurakulov *et al.*, 2015 ▸). Crystals suitable for X-ray diffraction were obtained from a solution in methanol by slow evaporation of the solvent at room temperature.

## Refinement details   

Crystal data, data collection parameters and refinement results are summarized in Table 2 ▸. H atoms on C atoms were positioned geometrically and treated as riding on their parent atoms, with C—H = 0.95 (aromatic), 0.98 (meth­yl), 0.99 (methyl­ene) or 1.00 Å (tertiary C atom) and were refined with *U*_iso_(H) = 1.5*U*_eq_(C) for methyl H atoms and 1.2*U*_eq_(C) otherwise. The H atom in the hy­droxy group of the co-crystallized methanol was refined with a distance restraint [target distance O—H = 0.84 (2) Å] and with *U*_iso_(H) = 1.2*U*_eq_(O). The anisotropic displacement parameters of N1 and O3 atom were subjected to an enhanced rigid-bond restraint (Thorn *et al.*, 2012 ▸).

## Supplementary Material

Crystal structure: contains datablock(s) I, test. DOI: 10.1107/S2056989020006696/fy2144sup1.cif

Structure factors: contains datablock(s) I. DOI: 10.1107/S2056989020006696/fy2144Isup2.hkl

Supporting information file. DOI: 10.1107/S2056989020006696/fy2144Isup3.cml

CCDC reference: 2004621

Additional supporting information:  crystallographic information; 3D view; checkCIF report

## Figures and Tables

**Table 1:** Hydrogen-bond geometry (Å, °)

*D*—H⋯*A*	*D*—H	H⋯*A*	*D*⋯*A*	*D*—H⋯*A*
O6—H6⋯O5	0.96	1.91	2.8581 (7)	171
C1—H1⋯O1^i^	1.00	2.55	3.4040 (8)	143
C1—H1⋯O2^i^	1.00	2.37	3.2444 (8)	146
C4—H4*A*⋯O5^ii^	0.99	2.45	3.4346 (8)	172
C15—H15⋯O1^iii^	0.95	2.44	3.3402 (8)	159
C16—H16⋯O2^iii^	0.95	2.59	3.3246 (8)	134
C25—H25*A*⋯O4^iv^	0.99	2.29	3.1224 (8)	141

Symmetry codes: (i) 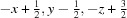; (ii) 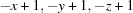; (iii) 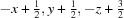; (iv) 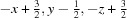.

**Table 2:** Experimental details

Crystal data	
Chemical formula	C_29_H_26_N_4_O_5_·CH_4_O
*M*_r_	542.58
Crystal system, space group	Monoclinic, *P*2_1_/*n*
Temperature (K)	100
*a*, *b*, *c* (Å)	16.326 (4), 8.0566 (19), 20.565 (5)
β (°)	104.497 (6)
*V* (Å^3^)	2618.9 (11)
*Z*	4
Radiation type	Mo *K*α
μ (mm^−1^)	0.10
Crystal size (mm)	0.55 × 0.09 × 0.08

Data collection	
Diffractometer	Bruker *APEX* CCD
Absorption correction	Multi-scan (*SADABS*; Bruker, 2008 ▸)
*T*_min_, *T*_max_	0.665, 0.745
No. of measured, independent and observed [*I* > 2σ(*I*)] reflections	25889, 4821, 2918
*R*_int_	0.114
(sin θ/λ)_max_ (Å^−1^)	0.604

Refinement	
*R*[*F* ^2^ > 2σ(*F* ^2^)], *wR*(*F* ^2^), *S*	0.061, 0.164, 1.04
No. of reflections	4821
No. of parameters	367
No. of restraints	4
H-atom treatment	H atoms treated by a mixture of independent and constrained refinement
Δρ_max_, Δρ_min_ (e Å^−3^)	0.39, −0.35

Computer programs: *APEX2* (Bruker, 2001 ▸), *SAINT-Plus* (Bruker, 2009 ▸), *SHELXT* (Sheldrick, 2015 ▸), *SHELXL2014/7* (Sheldrick, 2015 ▸), *PLATON* (Spek, 2020 ▸), *publCIF* (Westrip, 2010 ▸).

## supplementary crystallographic information

### Crystal data

**Table:**

C_29_H_26_N_4_O_5_·CH_4_O	*F*(000) = 1144
*M**_r_* = 542.58	*D*_x_ = 1.376 Mg m^−^^3^
Monoclinic, *P*2_1_/*n*	Mo *K*α radiation, λ = 0.71073 Å
*a* = 16.326 (4) Å	Cell parameters from 1353 reflections
*b* = 8.0566 (19) Å	θ = 3.0–19.8°
*c* = 20.565 (5) Å	µ = 0.10 mm^−^^1^
β = 104.497 (6)°	*T* = 100 K
*V* = 2618.9 (11) Å^3^	Rod, yellow
*Z* = 4	0.55 × 0.09 × 0.08 mm

### Data collection

**Table:**

Bruker APEX CCD diffractometer	4821 independent reflections
Radiation source: microsource	2918 reflections with *I* > 2σ(*I*)
Multilayer optics monochromator	*R*_int_ = 0.114
ω scans	θ_max_ = 25.4°, θ_min_ = 1.4°
Absorption correction: multi-scan (SADABS; Bruker, 2008)	*h* = −19→19
*T*_min_ = 0.665, *T*_max_ = 0.745	*k* = −9→9
25889 measured reflections	*l* = −24→24

### Refinement

**Table:**

Refinement on *F*^2^	4 restraints
Least-squares matrix: full	Hydrogen site location: mixed
*R*[*F*^2^ > 2σ(*F*^2^)] = 0.061	H atoms treated by a mixture of independent and constrained refinement
*wR*(*F*^2^) = 0.164	*w* = 1/[σ^2^(*F*_o_^2^) + (0.0677*P*)^2^ + 0.4688*P*] where *P* = (*F*_o_^2^ + 2*F*_c_^2^)/3
*S* = 1.04	(Δ/σ)_max_ < 0.001
4821 reflections	Δρ_max_ = 0.39 e Å^−^^3^
367 parameters	Δρ_min_ = −0.35 e Å^−^^3^

### Special details

**Table:**

Geometry. All esds (except the esd in the dihedral angle between two l.s. planes) are estimated using the full covariance matrix. The cell esds are taken into account individually in the estimation of esds in distances, angles and torsion angles; correlations between esds in cell parameters are only used when they are defined by crystal symmetry. An approximate (isotropic) treatment of cell esds is used for estimating esds involving l.s. planes.

### Fractional atomic coordinates and isotropic or equivalent isotropic displacement parameters (Å^2^)

**Table:**

	*x*	*y*	*z*	*U*_iso_*/*U*_eq_	
O1	0.08305 (12)	1.1111 (2)	0.65060 (10)	0.0267 (5)	
O2	0.14565 (12)	1.0007 (2)	0.77066 (9)	0.0256 (5)	
O4	0.71000 (15)	1.2323 (3)	0.94869 (12)	0.0522 (7)	
O3	0.60907 (14)	1.4105 (3)	0.94156 (11)	0.0385 (6)	
O5	0.70612 (13)	0.3657 (3)	0.50686 (10)	0.0303 (5)	
N1	0.63699 (17)	1.2806 (4)	0.92532 (13)	0.0338 (7)	
N2	0.46055 (15)	0.8631 (3)	0.66495 (12)	0.0238 (6)	
N19	0.59083 (15)	0.4135 (3)	0.66290 (12)	0.0260 (6)	
N24A	0.62119 (15)	0.5026 (3)	0.56116 (12)	0.0241 (6)	
C1	0.42173 (18)	0.8773 (4)	0.72189 (14)	0.0221 (7)	
H1	0.4208	0.7639	0.7415	0.026*	
C3	0.44128 (19)	1.0014 (4)	0.61757 (15)	0.0266 (7)	
H3B	0.4754	0.9925	0.5841	0.032*	
H3A	0.4551	1.1081	0.6418	0.032*	
C4	0.34774 (18)	0.9951 (4)	0.58261 (14)	0.0269 (7)	
H4B	0.3321	1.0936	0.5534	0.032*	
H4A	0.3356	0.8947	0.5540	0.032*	
C4A	0.29609 (19)	0.9915 (4)	0.63407 (14)	0.0236 (7)	
C5	0.21208 (19)	1.0482 (4)	0.61679 (15)	0.0248 (7)	
H5	0.1879	1.0853	0.5723	0.030*	
C6	0.16455 (18)	1.0508 (4)	0.66287 (15)	0.0237 (7)	
C7	0.19881 (18)	0.9950 (3)	0.72862 (14)	0.0226 (7)	
C8	0.28131 (19)	0.9378 (3)	0.74592 (14)	0.0226 (7)	
H8	0.3048	0.8982	0.7902	0.027*	
C8A	0.33064 (18)	0.9374 (3)	0.69924 (14)	0.0213 (7)	
C9	0.0470 (2)	1.1711 (4)	0.58366 (15)	0.0331 (8)	
H9B	0.0804	1.2652	0.5743	0.050*	
H9C	−0.0114	1.2071	0.5798	0.050*	
H9A	0.0474	1.0821	0.5513	0.050*	
C10	0.1823 (2)	0.9721 (4)	0.84092 (15)	0.0346 (9)	
H10B	0.2047	0.8588	0.8474	0.052*	
H10C	0.1389	0.9865	0.8659	0.052*	
H10A	0.2283	1.0515	0.8574	0.052*	
C11	0.47684 (18)	0.9873 (4)	0.77600 (14)	0.0213 (7)	
C12	0.55019 (19)	0.9188 (4)	0.81757 (15)	0.0279 (8)	
H12	0.5636	0.8057	0.8123	0.033*	
C13	0.6034 (2)	1.0145 (4)	0.86629 (15)	0.0297 (8)	
H13	0.6534	0.9687	0.8945	0.036*	
C14	0.58186 (19)	1.1787 (4)	0.87291 (15)	0.0258 (7)	
C15	0.51115 (19)	1.2512 (4)	0.83252 (14)	0.0262 (7)	
H15	0.4986	1.3649	0.8375	0.031*	
C16	0.45834 (19)	1.1526 (4)	0.78389 (14)	0.0256 (7)	
H16	0.4087	1.1997	0.7556	0.031*	
C17	0.50221 (18)	0.7246 (4)	0.65647 (15)	0.0223 (7)	
H17	0.5061	0.6442	0.6909	0.027*	
C18	0.53988 (18)	0.6787 (4)	0.60744 (15)	0.0238 (7)	
C18A	0.58472 (18)	0.5214 (4)	0.61466 (14)	0.0223 (7)	
C19A	0.64138 (18)	0.2763 (4)	0.66009 (15)	0.0239 (7)	
C20	0.6513 (2)	0.1578 (4)	0.71145 (16)	0.0303 (8)	
H20	0.6216	0.1704	0.7454	0.036*	
C21	0.7038 (2)	0.0237 (4)	0.71301 (17)	0.0381 (9)	
H21	0.7100	−0.0558	0.7480	0.046*	
C22	0.7478 (2)	0.0034 (5)	0.66366 (18)	0.0440 (10)	
H22	0.7848	−0.0886	0.6657	0.053*	
C23	0.7382 (2)	0.1152 (4)	0.61231 (17)	0.0343 (8)	
H23	0.7675	0.0995	0.5783	0.041*	
C23A	0.68515 (18)	0.2530 (4)	0.60979 (15)	0.0258 (7)	
C24	0.67353 (18)	0.3723 (4)	0.55492 (15)	0.0250 (7)	
C25	0.59868 (19)	0.6360 (4)	0.51134 (15)	0.0279 (8)	
H25B	0.5610	0.5943	0.4690	0.033*	
H25A	0.6499	0.6847	0.5014	0.033*	
C26	0.55278 (19)	0.7643 (4)	0.54499 (14)	0.0262 (7)	
H26A	0.5876	0.8656	0.5571	0.031*	
H26B	0.4978	0.7955	0.5146	0.031*	
C27	0.8885 (3)	0.3496 (6)	0.4479 (2)	0.0611 (12)	
H27A	0.9489	0.3688	0.4521	0.092*	
H27B	0.8599	0.3294	0.4007	0.092*	
H27C	0.8814	0.2527	0.4748	0.092*	
O6	0.85271 (16)	0.4911 (3)	0.47115 (13)	0.0523 (7)	
H6	0.8044 (18)	0.458 (5)	0.4864 (18)	0.063*	

### Atomic displacement parameters (Å^2^)

**Table:**

	*U*^11^	*U*^22^	*U*^33^	*U*^12^	*U*^13^	*U*^23^
O1	0.0260 (12)	0.0294 (13)	0.0210 (11)	0.0026 (10)	−0.0010 (9)	0.0009 (10)
O2	0.0272 (12)	0.0292 (13)	0.0193 (11)	0.0022 (10)	0.0034 (9)	0.0007 (10)
O4	0.0356 (15)	0.0495 (17)	0.0555 (17)	−0.0046 (13)	−0.0185 (13)	0.0101 (14)
O3	0.0387 (14)	0.0441 (16)	0.0304 (13)	−0.0057 (12)	0.0045 (11)	−0.0113 (12)
O5	0.0311 (12)	0.0356 (14)	0.0269 (12)	−0.0003 (10)	0.0123 (10)	−0.0025 (11)
N1	0.0309 (17)	0.0416 (19)	0.0256 (16)	−0.0090 (14)	0.0007 (13)	0.0040 (14)
N2	0.0272 (14)	0.0218 (15)	0.0218 (14)	0.0028 (12)	0.0054 (11)	0.0016 (12)
N19	0.0301 (15)	0.0237 (15)	0.0247 (15)	0.0019 (12)	0.0076 (12)	0.0005 (12)
N24A	0.0232 (14)	0.0241 (15)	0.0235 (14)	0.0000 (12)	0.0032 (11)	0.0004 (12)
C1	0.0255 (16)	0.0211 (17)	0.0191 (16)	0.0013 (13)	0.0047 (13)	0.0037 (14)
C3	0.0306 (18)	0.0245 (19)	0.0249 (17)	0.0010 (15)	0.0070 (14)	0.0035 (15)
C4	0.0306 (18)	0.0263 (19)	0.0219 (17)	−0.0025 (15)	0.0029 (14)	−0.0001 (15)
C4A	0.0318 (18)	0.0168 (17)	0.0211 (17)	−0.0018 (14)	0.0047 (14)	−0.0020 (14)
C5	0.0304 (18)	0.0218 (18)	0.0185 (16)	−0.0018 (14)	−0.0012 (14)	−0.0003 (14)
C6	0.0239 (17)	0.0189 (17)	0.0235 (17)	−0.0003 (14)	−0.0029 (14)	0.0006 (14)
C7	0.0268 (18)	0.0162 (16)	0.0233 (17)	−0.0028 (14)	0.0036 (14)	−0.0006 (14)
C8	0.0331 (18)	0.0146 (16)	0.0168 (16)	−0.0017 (14)	0.0002 (14)	−0.0009 (13)
C8A	0.0230 (16)	0.0158 (16)	0.0213 (17)	−0.0038 (13)	−0.0016 (13)	−0.0025 (13)
C9	0.0310 (18)	0.039 (2)	0.0235 (18)	0.0068 (16)	−0.0032 (15)	−0.0002 (16)
C10	0.039 (2)	0.043 (2)	0.0218 (18)	0.0109 (17)	0.0081 (15)	0.0058 (16)
C11	0.0216 (16)	0.0244 (18)	0.0172 (16)	−0.0012 (13)	0.0037 (13)	0.0020 (14)
C12	0.0292 (18)	0.0254 (19)	0.0283 (18)	0.0042 (15)	0.0061 (15)	0.0049 (15)
C13	0.0260 (18)	0.032 (2)	0.0265 (18)	−0.0008 (15)	−0.0017 (14)	0.0081 (16)
C14	0.0252 (17)	0.030 (2)	0.0207 (17)	−0.0049 (14)	0.0021 (14)	−0.0015 (15)
C15	0.0281 (18)	0.0268 (19)	0.0236 (17)	0.0010 (15)	0.0063 (15)	−0.0001 (15)
C16	0.0257 (17)	0.0284 (19)	0.0214 (17)	−0.0017 (15)	0.0034 (14)	0.0014 (15)
C17	0.0230 (16)	0.0179 (17)	0.0235 (17)	−0.0009 (13)	0.0011 (14)	0.0011 (14)
C18	0.0225 (16)	0.0240 (18)	0.0242 (17)	−0.0027 (13)	0.0044 (14)	−0.0005 (14)
C18A	0.0199 (16)	0.0237 (18)	0.0226 (17)	−0.0038 (13)	0.0038 (13)	−0.0040 (14)
C19A	0.0203 (16)	0.0202 (18)	0.0290 (18)	−0.0054 (13)	0.0023 (14)	−0.0048 (15)
C20	0.0304 (18)	0.033 (2)	0.0269 (18)	0.0010 (16)	0.0062 (15)	−0.0002 (16)
C21	0.043 (2)	0.037 (2)	0.035 (2)	0.0123 (17)	0.0106 (17)	0.0080 (17)
C22	0.048 (2)	0.042 (2)	0.043 (2)	0.0239 (19)	0.0138 (19)	0.0072 (19)
C23	0.037 (2)	0.034 (2)	0.034 (2)	0.0098 (16)	0.0120 (16)	0.0017 (17)
C23A	0.0213 (16)	0.0265 (18)	0.0282 (18)	0.0010 (14)	0.0036 (14)	−0.0012 (15)
C24	0.0207 (16)	0.0246 (18)	0.0279 (18)	−0.0036 (14)	0.0025 (14)	−0.0063 (15)
C25	0.0280 (17)	0.032 (2)	0.0231 (17)	−0.0015 (15)	0.0052 (14)	0.0017 (15)
C26	0.0265 (17)	0.0252 (18)	0.0260 (18)	−0.0002 (14)	0.0049 (14)	0.0027 (15)
C27	0.060 (3)	0.072 (3)	0.048 (3)	0.019 (2)	0.007 (2)	−0.011 (2)
O6	0.0492 (17)	0.0592 (19)	0.0511 (17)	−0.0084 (14)	0.0174 (14)	−0.0151 (14)

### Geometric parameters (Å, º)

**Table:**

O1—C6	1.379 (3)	C10—H10C	0.9800
O1—C9	1.439 (4)	C10—H10A	0.9800
O2—C7	1.371 (3)	C11—C16	1.384 (4)
O2—C10	1.438 (3)	C11—C12	1.399 (4)
O4—N1	1.232 (3)	C12—C13	1.384 (4)
O3—N1	1.221 (3)	C12—H12	0.9500
O5—C24	1.236 (3)	C13—C14	1.384 (4)
N1—C14	1.470 (4)	C13—H13	0.9500
N2—C17	1.341 (4)	C14—C15	1.373 (4)
N2—C3	1.462 (4)	C15—C16	1.394 (4)
N2—C1	1.469 (3)	C15—H15	0.9500
N19—C18A	1.304 (4)	C16—H16	0.9500
N19—C19A	1.389 (4)	C17—C18	1.357 (4)
N24A—C24	1.379 (4)	C17—H17	0.9500
N24A—C18A	1.384 (3)	C18—C18A	1.452 (4)
N24A—C25	1.467 (4)	C18—C26	1.518 (4)
C1—C8A	1.522 (4)	C19A—C20	1.403 (4)
C1—C11	1.527 (4)	C19A—C23A	1.409 (4)
C1—H1	1.0000	C20—C21	1.374 (4)
C3—C4	1.517 (4)	C20—H20	0.9500
C3—H3B	0.9900	C21—C22	1.393 (4)
C3—H3A	0.9900	C21—H21	0.9500
C4—C4A	1.510 (4)	C22—C23	1.367 (5)
C4—H4B	0.9900	C22—H22	0.9500
C4—H4A	0.9900	C23—C23A	1.401 (4)
C4A—C8A	1.388 (4)	C23—H23	0.9500
C4A—C5	1.404 (4)	C23A—C24	1.458 (4)
C5—C6	1.367 (4)	C25—C26	1.539 (4)
C5—H5	0.9500	C25—H25B	0.9900
C6—C7	1.402 (4)	C25—H25A	0.9900
C7—C8	1.383 (4)	C26—H26A	0.9900
C8—C8A	1.399 (4)	C26—H26B	0.9900
C8—H8	0.9500	C27—O6	1.418 (4)
C9—H9B	0.9800	C27—H27A	0.9800
C9—H9C	0.9800	C27—H27B	0.9800
C9—H9A	0.9800	C27—H27C	0.9800
C10—H10B	0.9800	O6—H6	0.957 (18)
			
C6—O1—C9	116.0 (2)	C13—C12—H12	119.7
C7—O2—C10	117.3 (2)	C11—C12—H12	119.7
O3—N1—O4	123.7 (3)	C14—C13—C12	118.4 (3)
O3—N1—C14	118.5 (3)	C14—C13—H13	120.8
O4—N1—C14	117.7 (3)	C12—C13—H13	120.8
C17—N2—C3	125.4 (2)	C15—C14—C13	122.9 (3)
C17—N2—C1	120.2 (2)	C15—C14—N1	118.3 (3)
C3—N2—C1	114.1 (2)	C13—C14—N1	118.8 (3)
C18A—N19—C19A	115.6 (2)	C14—C15—C16	117.8 (3)
C24—N24A—C18A	123.9 (3)	C14—C15—H15	121.1
C24—N24A—C25	123.0 (2)	C16—C15—H15	121.1
C18A—N24A—C25	113.1 (2)	C11—C16—C15	121.2 (3)
N2—C1—C8A	111.4 (2)	C11—C16—H16	119.4
N2—C1—C11	109.5 (2)	C15—C16—H16	119.4
C8A—C1—C11	113.0 (2)	N2—C17—C18	131.5 (3)
N2—C1—H1	107.6	N2—C17—H17	114.3
C8A—C1—H1	107.6	C18—C17—H17	114.3
C11—C1—H1	107.6	C17—C18—C18A	118.2 (3)
N2—C3—C4	108.2 (2)	C17—C18—C26	133.6 (3)
N2—C3—H3B	110.0	C18A—C18—C26	108.0 (2)
C4—C3—H3B	110.0	N19—C18A—N24A	124.4 (3)
N2—C3—H3A	110.0	N19—C18A—C18	126.9 (3)
C4—C3—H3A	110.0	N24A—C18A—C18	108.7 (3)
H3B—C3—H3A	108.4	N19—C19A—C20	117.9 (3)
C4A—C4—C3	109.9 (2)	N19—C19A—C23A	123.5 (3)
C4A—C4—H4B	109.7	C20—C19A—C23A	118.5 (3)
C3—C4—H4B	109.7	C21—C20—C19A	120.4 (3)
C4A—C4—H4A	109.7	C21—C20—H20	119.8
C3—C4—H4A	109.7	C19A—C20—H20	119.8
H4B—C4—H4A	108.2	C20—C21—C22	120.5 (3)
C8A—C4A—C5	118.8 (3)	C20—C21—H21	119.7
C8A—C4A—C4	121.3 (3)	C22—C21—H21	119.7
C5—C4A—C4	119.9 (3)	C23—C22—C21	120.3 (3)
C6—C5—C4A	121.2 (3)	C23—C22—H22	119.8
C6—C5—H5	119.4	C21—C22—H22	119.8
C4A—C5—H5	119.4	C22—C23—C23A	120.1 (3)
C5—C6—O1	124.6 (3)	C22—C23—H23	120.0
C5—C6—C7	120.3 (3)	C23A—C23—H23	120.0
O1—C6—C7	115.0 (3)	C23—C23A—C19A	120.1 (3)
O2—C7—C8	125.3 (3)	C23—C23A—C24	120.7 (3)
O2—C7—C6	115.8 (3)	C19A—C23A—C24	119.2 (3)
C8—C7—C6	118.9 (3)	O5—C24—N24A	120.5 (3)
C7—C8—C8A	121.0 (3)	O5—C24—C23A	126.3 (3)
C7—C8—H8	119.5	N24A—C24—C23A	113.2 (3)
C8A—C8—H8	119.5	N24A—C25—C26	104.1 (2)
C4A—C8A—C8	119.8 (3)	N24A—C25—H25B	110.9
C4A—C8A—C1	121.7 (3)	C26—C25—H25B	110.9
C8—C8A—C1	118.5 (3)	N24A—C25—H25A	110.9
O1—C9—H9B	109.5	C26—C25—H25A	110.9
O1—C9—H9C	109.5	H25B—C25—H25A	109.0
H9B—C9—H9C	109.5	C18—C26—C25	105.2 (2)
O1—C9—H9A	109.5	C18—C26—H26A	110.7
H9B—C9—H9A	109.5	C25—C26—H26A	110.7
H9C—C9—H9A	109.5	C18—C26—H26B	110.7
O2—C10—H10B	109.5	C25—C26—H26B	110.7
O2—C10—H10C	109.5	H26A—C26—H26B	108.8
H10B—C10—H10C	109.5	O6—C27—H27A	109.5
O2—C10—H10A	109.5	O6—C27—H27B	109.5
H10B—C10—H10A	109.5	H27A—C27—H27B	109.5
H10C—C10—H10A	109.5	O6—C27—H27C	109.5
C16—C11—C12	119.1 (3)	H27A—C27—H27C	109.5
C16—C11—C1	122.5 (3)	H27B—C27—H27C	109.5
C12—C11—C1	118.3 (3)	C27—O6—H6	109 (2)
C13—C12—C11	120.5 (3)		

### Hydrogen-bond geometry (Å, º)

**Table:**

*D*—H···*A*	*D*—H	H···*A*	*D*···*A*	*D*—H···*A*
O6—H6···O5	0.96	1.91	2.8581 (7)	171
C1—H1···O1^i^	1.00	2.55	3.4040 (8)	143
C1—H1···O2^i^	1.00	2.37	3.2444 (8)	146
C4—H4*A*···O5^ii^	0.99	2.45	3.4346 (8)	172
C9—H9*B*···O6^iii^	0.98	2.54	3.5042 (9)	169
C15—H15···O1^iv^	0.95	2.44	3.3402 (8)	159
C16—H16···O2^iv^	0.95	2.59	3.3246 (8)	134
C17—H17···N19	0.95	2.47	2.8805 (7)	106
C25—H25*A*···O4^v^	0.99	2.29	3.1224 (8)	141

Symmetry codes: (i) −*x*+1/2, *y*−1/2, −*z*+3/2; (ii) −*x*+1, −*y*+1, −*z*+1; (iii) −*x*+1, −*y*, −*z*+1; (iv) −*x*+1/2, *y*+1/2, −*z*+3/2; (v) −*x*+3/2, *y*−1/2, −*z*+3/2.
